# The effect of feed quality due to clarification strategy on the design and performance of protein A periodic counter‐current chromatography

**DOI:** 10.1002/btpr.2709

**Published:** 2018-10-03

**Authors:** Hani El‐Sabbahy, David Ward, Olotu Ogonah, Lynne Deakin, Gregory M. Jellum, Daniel G. Bracewell

**Affiliations:** ^1^ Present address: Separation and Purification Sciences Div. 3M United Kingdom PLC, 3M Centre Bracknell RG12 8HT U.K.; ^2^ Dept. of Biochemical Engineering University College London London WC1E 6BT U.K.; ^3^ Dept. of Biochemical Engineering University College London London WC1E 6BT U.K.; ^4^ Separation and Purification Sciences Div. 3M United Kingdom PLC, 3M Centre Bracknell RG12 8HT U.K.; ^5^ Separation and Purification Sciences Div. 3M Centre St. Paul MN 55144; ^6^ Dept. of Biochemical Engineering University College London London WC1E 6BT UK

**Keywords:** productivity, purity, protein A, clarification, periodic counter‐current chromatography

## Abstract

The impact of two different quality feeds, derived using two different harvest clarification processes, on protein A periodic counter‐current chromatography (PCC) design and performance is investigated. Data from batch experiments were input into a model to design optimal PCC operating parameters specific to each feed material. The two clarification methods were: depth filtration using a wetlaid matrix which has Q‐functionality; and a combination of depth filtration and chromatographic clarification, using a Q‐functional nonwoven with a high anion exchange capacity (Emphaze™ AEX Hybrid Purifier) in which key impurities such as host cell DNA (HCDNA) and host cell proteins (HCP) are removed. The model predicted 34% better productivity for the chromatographically clarified cell culture fluid (CCCF) using a 4 column system, and productivity gains of 28% using only 3 columns enabling the option to simplify the protein A PCC strategy. Experimental validation of the predicted optimized PCC operating parameters using industrially relevant monoclonal antibody (mAb) CCCF feedstock over 100 cycles showed productivity gains of 49% for the chromatographically clarified material. HCP concentration was 11‐fold lower, and HCDNA concentration was reduced by 4.4 Log Reduction Value (LRV) in the protein A PCC eluates. This work, therefore, demonstrates that the removal of HCDNA and HCP during clarification is an effective strategy for improving protein A PCC performance. This was achieved using the Emphaze™ AEX Hybrid Purifier which can be easily incorporated into a batch or continuous process, in a scalable fashion, without adding additional separate unit operations. © 2018 American Institute of Chemical Engineers *Biotechnol. Prog*., 34:1380–1392, 2018

## Introduction

Continuous processing has the potential to bring many advantages to the production of biotherapeutics such as lower capital costs, due to the use of smaller equipment and storage vessels, higher productivity, lower buffer consumption, and the possibility of steady‐state operation in some circumstances.^1^, ^2^, ^3^

A number of academic and commercial entities are currently exploring continuous processing for recombinant protein manufacturing.^4^ However, adoption has been slow due to the lack of a regulatory precedent, the need for better online process analytics (PAT) to improve process control, the inertia due to existing capital equipment for batch processing, and the perceived complexity associated with continuous manufacturing.^3^, ^5^, ^6^, ^7^, ^8^

A significant amount of work has been done by different research groups on periodic counter‐current chromatography (PCC), and several groups have demonstrated capacity utilization and productivity gains over batch systems in most cases.^9^, ^10^, ^11^, ^12^ There has also been some work, using decisional tools, which demonstrates the economic benefits of inclusion of continuous capture chromatography as part of a hybrid approach for commercial production.^13^ However, as with continuous processing in general, one of the barriers to implementation of continuous multicolumn chromatography is system complexity, particularly, when more than 4 columns are used.^14^

Higher titer feed materials generally tend to increase system complexity. This is because, as the load time becomes shorter, there is a requirement for more columns to maintain the balance between loading and regeneration which is the key to maximizing productivity.^15^ The alternative is to reduce the regeneration time by pre‐treating the feed material to remove host cell protein (HCP) and host cell DNA (HCDNA). This may enable further optimization of the regeneration steps and allow fewer columns to be used, reducing system complexity.^3^ In this work this hypothesis is tested.

Different modeling and design approaches have been proposed by various research groups.^9^, ^10^, ^16^, ^17^, ^18^ The majority of these are complex and therefore not simple to implement. The model proposed by Gjoka et al. is much less complex, but requires a nonstandard column setup, that is, two columns in series with two UV detectors. Also, while productivity and capture efficiency predictions were made, only the capture efficiency appears to have been validated experimentally. In this work, a simple methodology using standard batch chromatography equipment for the design and optimization of a protein A PCC capture step for maximum productivity is presented. Experimental validation of the results using industry relevant CHO expressed monoclonal antibody (mAb) CCCF is provided.

Chromatographic clarification, using Q‐functional nonwoven technology, has been shown to result in levels of HCDNA 3.5 logs lower and HCP 19 times lower in the protein A column eluate, compared to material clarified by depth filter alone.^19^ The Emphaze™ AEX Hybrid Purifier enables reduction of soluble and insoluble contaminants by charge using a Q‐functional nonwoven matrix. It consists of four layers of quaternary ammonium functionalized nonwoven followed by an asymmetric polyamide membrane with a final pore size of 0.2 μm. Due to the open media structure and high anion exchange capacity, it is able to handle significant turbidity while removing soluble impurities. This work aims to investigate whether similar improvements in post protein A purity can also be seen post protein A PCC.

Protein A performance was compared using single column, single cycle, batch runs on control (depth filtered) and chromatographically clarified CCCF. The batch data were then used to design a PCC unit operation optimized for each feed material using a simple model. The design was evaluated experimentally by conducting 100 cycles of PCC. The experimentally obtained performance with each feed material was then compared to investigate the impact of feed quality on the performance of the PCC unit operation in terms of productivity, capacity utilization, and purity.

## Theoretical Considerations

### *PCC theory*

The steps required for a batch chromatography unit operation include equilibration of the chromatography column, loading, post‐load wash, elution, column cleaning, and re‐equilibration. The same steps are required in a PCC unit operation, but they are divided into two groups—loading and regeneration (which includes the post‐load wash, elution, cleaning, and re‐equilibration).

In addition to these steps, the PCC unit operation will have two or more columns in the loading zone. This enables the capacity of the first chromatography column to be more fully utilized by capturing product which flows through when it is overloaded. It is this greater utilization of capacity that leads to lower buffer consumption and smaller column size.^17^

The three key steps in a PCC unit operation are feed, flow‐through loading, and regeneration. Tables 1, 2, 3 show the order of these steps in a single cycle on a 2, 3, and 4 column PCC system. The flowthrough loading is referred to simply as flowthrough in these tables. From the tables, it can be seen that each column is subject to each of the steps described above, but with different steps happening in parallel on different columns. It is this parallel operation that contributes to the productivity gains compared to batch processing.

**Table 1:** Table Showing the Steps in a PCC Cycle for a System Containing Two Columns

Step	Column 1	Column 2
1	Feed	Regeneration
2	Feed	Flowthrough
3	Regeneration	Feed
4	Flowthrough	Feed

**Table 2:** Table Showing the Steps in a PCC Cycle for a System Containing Three Columns

Step	Column 1	Column 2	Column 3
1	Feed	Flowthrough	Regeneration
2	Regeneration	Feed	Flowthrough
3	Flowthrough	Regeneration	Feed

**Table 3:** Table Showing the Steps in a PCC Cycle for a System Containing Four Columns

Step	Column 1	Column 2	Column 3	Column 4
1	Feed	Flowthrough	Regeneration 2	Regeneration 1
2	Regeneration 1	Feed	Flowthrough	Regeneration 2
3	Regeneration 2	Regeneration 1	Feed	Flowthrough
4	Flowthrough	Regeneration 2	Regeneration 1	Feed

Changing the number of columns changes the relative length of the feed and regeneration steps with feed being longer than regeneration for a 2 column system, feed being the same as regeneration for a 3 column system, and feed being shorter than the regeneration for a 4 column system.

### *Model*

A model to predict the productivity of a PCC chromatography unit operation was developed for 2, 3, and 4 column systems building on previously published work^9^, ^20^. The maximum number of columns was limited to four to minimize system complexity. However, the model is adaptable for more columns.

The underlying assumptions of the model are as follows:

- All mass that is not bound by the feed column (*M*_ub_) is bound by the flowthrough column.
- This model ignores any kinetic effects due to the different concentration that is loaded onto a fresh column in the first load step of the first cycle (*C*_0_) and the variable concentration that is loaded onto the flowthrough column during all other cycles (*C*_FT_).
- Breakthrough curve shape is identical between the first cycle and the *n*th cycle.
- All bound product is recovered from the model during the elution.

The productivity (*P*) is defined as the total mass, in grams, bound (*M*_*T*_) per unit volume of chromatography resin, in liters, (*V*_*R*_) per unit run time, in hours, (*t*_*T*_) as described by Eq. (1)

(1) $$P=\frac{M_{T}}{V_{R}t_{T}}$$

**Figure 1:**
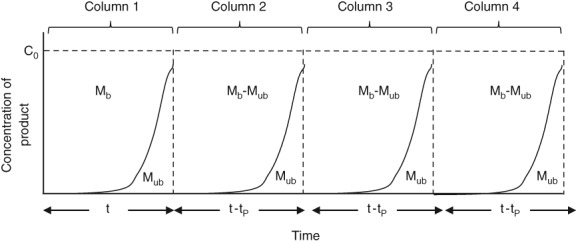
Diagram of theoretical breakthrough curves for a PCC run with four columns. *M*_b_ is the mass of protein bound to column. *M*_ub_ is the quantity of protein that flows through the first column in the loading zone and binds to the second column and *t*_*p*_ is the preload time, which is the time required to load a quantity of product equivalent to *M*_ub_.

Figure 1 shows theoretical breakthrough curves for a single cycle of a 4 column PCC run. The load on the first column, during the first cycle, is the longest as it has not been preloaded with any product. For the second, third and fourth column, and subsequent cycles, the length of the load is reduced by *t*_*p*_ which is the length of time required to load a quantity of feed material containing the same amount of product as that which flows through the preceding column, that is, *M*_ub_. This is because each of these columns will already have been exposed to a preload equal to *M*_ub_.

As it is assumed that all product either binds to the feed or flowthrough column, the sum of the area above and below each of these curves will give rise to the total quantity of product bound. This gives rise to Eq. (2), where *M*_*b*_ is the quantity of bound protein, *N*_*c*_ is the number of columns, and *X* is the number of cycles

(2) $$M_{T}=XN_{c}M_{b}+M_{\mathrm{ub}}$$

The unbound mass is evaluated from the area under a single column breakthrough curve up to the desired level of product breakthrough. This is expressed in Eq. (3), where *Q* is the volumetric flowrate of the feed, *C*_FT_(*t*) is the concentration of product in the column flowthrough as a function of time, and *t* is the time at which the desired percentage breakthrough is achieved. When selecting the desired percentage breakthrough, for PCC configurations where there are two columns in the loading zone, the unbound mass should not be larger than the amount of product that would be loaded under batch operations (*t*_DBC_) as this would lead to product loss. For the purpose of this work, this value was less than or equal to the mass of antibody contained in 80% of the volume at which 5% breakthrough is achieved

(3) $$M_{\mathrm{ub}}=Q\int_{0}^{t}C_{\text{FT}}\left(t\right)\mathrm{dt}$$

The bound mass is evaluated from the area above a single column breakthrough curve as expressed in Eq. (4), where *C*_0_ is the feed concentration

(4) $$M_{b}=C_{0}\mathrm{tQ}-M_{\mathrm{ub}}$$

The total run time *t*_*T*_ is expressed by Eq. (5), where *t*_*i*_ is the time required for equilibrating the first column in preparation to be loaded, *t*_*s*_ is the length of time at which the feed is switched to the next column, and *t*_RF_ is the regeneration time of any loaded columns after all cycles have been completed

(5) $$t_{T}=t_{i}+t_{p}+{\mathrm{XN}}_{c}t_{S}+t_{\mathrm{RF}}$$

The primary difference between the 2, 3, and 4 column models is in the regeneration time. To maximize throughput, the feed should be loading onto a column throughout the cycle. For a 2 column system, where the feed is loading onto a single column while regeneration is taking place, the regeneration time *t*_*R*_ should be shorter than the time taken for the antibody to begin breaking through the first column (*t*_DBC_) or antibody will be lost. These criteria are expressed in Eqs. (6)–(8), where *t*_*L*_ is the load time and *t*_BT‐DBC_ is the time between *t*_DBC_ and the desired percentage breakthrough

(6) $$t_{R}\leq t_{\mathrm{DBC}}$$

(7) $$t_{L}=t_{S}=t_{\mathrm{DBC}}+t_{\mathrm{BT}-\mathrm{DBC}}$$

(8) $$t_{L}=t-t_{p}$$

For 3 column systems, the regeneration time should simply be shorter than the loading time *t*_*L*,_ as expressed in Eqs. (9) and (10), otherwise the column will not be loading all the time which will reduce productivity

(9) $$t_{S}=t_{L}\;\text{when}\;t_{L}\geq t_{R}$$

(10) $$t_{S}=t_{R}\;\text{when}\;t_{L}<t_{R}$$

For the 4 column system, the regeneration step is split into two sections and occurs over two load steps. The total regeneration must again be complete within those two load steps otherwise there will be an interruption to loading which will reduce productivity as expressed in Eqs. (11) and (12)

(11) $$t_{S}=t_{L}\;\text{when}\;t_{L}\geq \frac{t_{R}}{2}$$

(12) $$t_{S}=\frac{t_{R}}{2}\;\text{when}\;t_{L}<\frac{t_{R}}{2}$$

**Figure 2:**
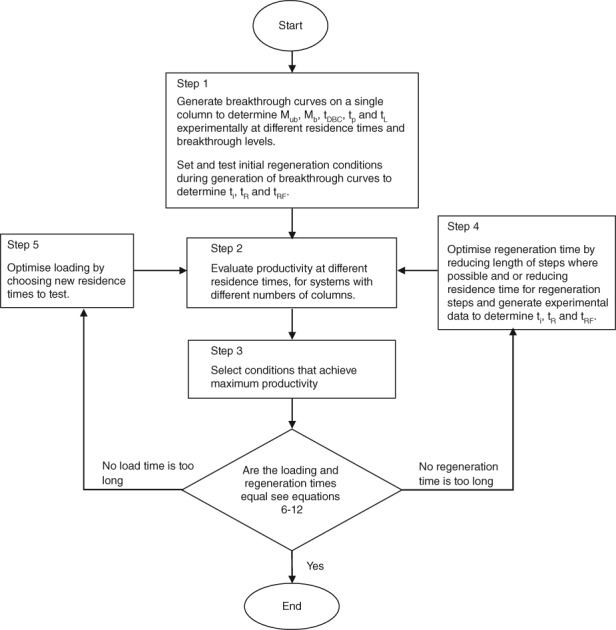
Process for using the PCC model to optimize for maximum productivity.

Figure 2 shows the process that was used to arrive at the operating conditions for the two feed materials used in this work. The PCC operating conditions for each feed material where arrived at independently of the other. All steps with the exception of Step 5 were undertaken in this study. While further optimization of residence time for the Emphaze™ AEX Hybrid Purifier feed material could have been achieved by generation of breakthrough curves at intermediate residence times, the conditions for the 90ZB08A feed material were near optimal. Further optimization of the Emphaze™ AEX Hybrid Purifier feed material was judged not to be necessary as a difference in productivity had already been demonstrated.

## Materials and Methods

### *Reagents*

Citric acid, sodium phosphate monobasic monohydrate, and sodium phosphate dibasic heptahydrate were purchased from Fisher Chemicals (Loughborough, U.K.). Glacial acetic acid was purchased from VWR (Lutterworth, U.K.). Sodium hydroxide pellets, sodium chloride, Tris‐hydrochloride, Tris‐base, sodium sulphate, sulphuric acid, hydrochloric acid, and glycine were purchased from Sigma‐Aldrich (Gillingham, U.K.).

### *Common buffers*

The following buffers were used routinely through this article and are defined here:

- Equilibration buffer: 20 mM sodium phosphate,150 mM NaCl, pH 7.0.
- Elution buffer: 100 mM citrate buffer, pH 3.2.
- Neutralization buffer: 1 M Tris–HCl buffer, pH 9.0.

All buffers were filtered through a LifeASSURE™ PDA020 0.2 μm sterilizing grade membrane filters (3M, St Paul, MN, USA) prior to use.

### *Preparation of feed for chromatography runs*

A tocilizumab biosimilar feed stock was produced in CHO cell culture. The material was produced in fed‐batch in two 50 L disposable stirred tank bioreactors (Eppendorf, Hamburg, Germany) up to cell densities of 5.7 × 10^6^ cells/mL and 6.6 × 10^6^ cells/mL, with final viabilities of 64% and 74%, respectively. The bioreactors were harvested on day 14 separately through a 30SP02A primary Zeta Plus™ depth filter (3M) at throughputs of 75 L/m^2^ and 78 L/m^2^, respectively, and a flux of 261 liters per meter square per hour (LMH).

The 30SP02A filtrates were then pooled and mixed. The antibody titer of this material was measured as described in the Analytics section of the Materials and Methods and was 3.5 g/L. A portion of the pool was further clarified through a 90ZB08A Zeta Plus™ polishing grade depth filter (3M) which contains *Q* functionality and is highly charged compared to other depth filters. A throughput of 243 L/m^2^ and flux of 197 LMH was achieved. It was then sterile filtered using a 0.2 μm LifeASSURE™ PDA membrane filter (3M) at a throughput of 207 L/m^2^ and flux of 168 LMH. This material was aliquoted and frozen at −80°C. It is referred to in this article as “depth filter clarified material,” and represents material clarified through a highly charged depth filter train.

A separate portion of the 30SP02A filtrate material was further clarified using the Emphaze™ AEX Hybrid Purifier, a flowthrough anion exchange chromatography product (FT‐AEX) able to handle turbidity (3M), at a throughput of 262 L/m^2^ and a flux of 197 LMH, and then through a 0.2 μm LifeASSURE™ PDA membrane filter (3M) at a throughput of 223 L/m^2^ and flux of 168 LMH. This material was also aliquoted and frozen at −80°C and is referred to in this article as “FT‐AEX clarified material.”

Feed material was thawed at room temperature prior to use. As no changes to turbidity or particulates were observed in either of the thawed feeds, filtration with a 0.2 μm membrane was not conducted prior to use. All measurements reported were conducted on the thawed material.

### *Single column breakthrough curves*

An ÄKTA™ Avant chromatography system (GE, Uppsala, Sweden) was used for all single column experiments. HiTrap MabSelect SuRe™ 1 mL columns (GE), with a bed height of 2.5 cm, were use. Separate columns were used for depth filter and FT‐AEX clarified feed materials. All samples were applied to the columns using the sample pump.

#### *Breakthrough Curves*

Breakthrough curves were developed by overloading the column with sample and analyzing the flowthrough samples for antibody concentration. The method steps, buffers, flow rates, and volumes are listed in Table 4. Fractions were collected every 0.5 mL from the start of feed loading, in a 96 well deepwell plate, until the end of sample application. The elution fraction peak was automatically collected in a 15 mL falcon tube when the UV 280 nm signal rose above 50 mAU. The tube was prepared with 0.42 mL neutralization buffer. The whole acid strip fraction was collected in a 15 mL falcon tube containing 1.2 mL neutralization buffer. All fractions were analyzed on a Protein G column as described in the Analytics section of the Materials and Methods.

**Table 4:** Process Steps, Buffers, Flow Rates, and Volumes for the Development of Single Column Breakthrough Curves

Step	Buffer/Sample	Flow Rate (mL/min)	Volume (mL)
Equilibration	Equilibration buffer	1	10
Feed	FT‐AEX or depth filter clarified feed	Variable	Variable
Post‐load wash	Equilibration buffer	1	10
Elution	Elution buffer	1	8
Acid strip	500 mM acetic acid	0.5	2
Equilibration	Equilibration buffer	1	10
Sanitisation	100 mM NaOH	0.3	4.5
Equilibration	Equilibration buffer	1	10

#### *Batch Run*

A low load volume batch run was performed by modifying the method described in the Breakthrough Curves section. The feed step was performed with the depth filter clarified feed material at a flow rate of 0.25 mL/min and a load volume of 9 mL. All other flow rates were modified to 0.5 mL/min with the exception of the sanitisation step which was kept at 0.3 mL/min. All other step volumes were kept constant. Fractionation was also modified with the feed step collected as a whole fraction and 0.84 mL of Tris neutralization buffer added to the elution fraction.

#### *Long Load Batch Run*

Specific single column batch experiments were run with conditions similar to those in the PCC operation. An extended feed step was performed based on the total load volume predicted by the PCC model (as described in the Model section of this article). This total load volume includes the preload volume used on the first column in PCC operation as described in the PCC Run Methods section, thus mimicking the first column in the first cycle of PCC operation. The steps performed, the flow rates and volumes for each feed material are shown in Table 5.

**Table 5:** Flow Rates and Volumes Used for Batch Separations with FT‐AEX and Depth Filter Clarified Feed Materials to Mimic Conditions in PCC Operation

Steps	FT‐AEX Clarified Feed		Depth Filter Clarified Feed	
	Flow Rate (mL/min)	Volume (mL)	Flow Rate (mL/min)	Volume (mL)
Equilibration	0.5	10	0.5	10
Feed	1.0	13.5	0.5	20
Post‐load wash	0.5	5	0.5	5
Elution	0.5	5	0.5	8
Acid strip	N/A	N/A	0.5	2
Equilibration	0.5	10	0.5	10

### *Periodic counter‐current chromatography*

#### *Octave™ 10 BioSMB Setup*

**Figure 3:**
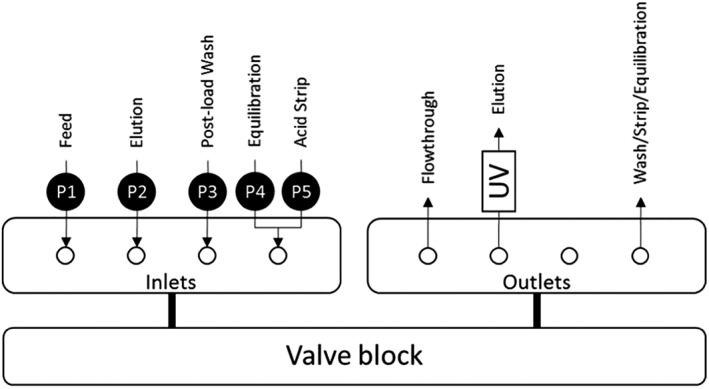
Schematic showing the Semba Octave 10 setup.

4 Column PCC (4C‐PCC) was performed on a Semba Octave™ 10 BioSMB (Semba Biosciences, Madison, WI, USA) with five pumps attached to the four inlet ports. The inlet ports feed to the valve block with four HiTrap MabSelect SuRe™ 1 mL columns, with a bed height of 2.5 cm, attached (GE). The valve block is controlled by Semba™ Pro software and allows control of the inlets and outlets of each column, including running columns in series. A schematic is shown in Figure 3.

The buffers used for each inlet are shown in Table 4. The Elution outlet was fed directly to a Semba Octave™ 4XE UV–VIS Detector (Semba Biosciences) fitted with a preparative flow cell (0.3 mm). Fractionation details are provided in Single Cycle PCC runs and 100 Cycle Experiments sections, of the Materials and Methods, for single cycle and 100 cycle PCC runs, respectively.

Throughout the 4C‐PCC, each column is one of seven positions with its own combination of inlets and outlets (see Table 6). The feed and flowthrough steps feature two columns in series, with the outlet of the feed column used as the inlet for the flowthrough column. The wash/strip/equilibration outlet contains the post‐load wash, acid strip, or equilibration steps combined into a single outlet.

**Table 6:** Column Inlets and Outlets for Each Position

Position	Inlet	Outlet
Flowthrough	From feed outlet	Flowthrough out
Feed	Feed in (P1)	To flowthrough in
Post‐load wash	Post‐load wash in (P3)	Wash/strip/equilibration out
Elution	Elution in (P2)	Elution out
Acid strip (depth filter clarified material only)	Acid strip in (P5)	Wash/strip/equilibration out
Equilibration	Equilibration in (P4)	Wash/strip/equilibration out
Rest	None	None

#### *PCC Run Methods*

Each of the two feed materials (depth filter and FT‐AEX clarified material) were run with modified methods, however, the base of the method contains the same three stages:

1. Preload: Initial loading of the feed and flowthrough columns with a small amount of feed.
2. Steady‐state cycles: The main loop, repeating for the number of cycles being run.
3. Post run: A final repeat of the steady‐state cycle without the feed and flowthrough positions to elute any remaining antibody from the columns.

The preload section features just the first two columns in series (feed and flowthrough) with the feed material being pumped for a set time. The preload time was calculated from the unbound area of the breakthrough curve as described in Model section of this article. This section was used to reduce the time required to reach steady state in continuous operation. The flow rate used was dependent on the feed material and set to the value used in the steady‐state cycles to ensure a consistent residence time between loading and preloading (0.5 mL/min for depth filter clarified feed material and 1 mL/min for FT‐AEX clarified feed material). The preload times were set to 5 min (2.5 mL) for depth filter clarified feed material and 3 min (3 mL) for FT‐AEX clarified feed material.

For the steady‐state cycles, all pump flow rates were run at 0.5 mL/min with the exception of the feed pump (P1) which was run at 1 mL/min for the FT AEX clarified feed and 0.5 mL/min for the depth filter clarified material. For the FT‐AEX method, the acid strip position was not used. Each steady‐state cycle was split into four switches (when the feed switches to the next column). Each of these was split into four steps; changing the post‐load wash, elution, acid strip, or equilibration positions of the two non‐loading columns. The steady‐state cycle steps for each column for the depth filter and FT‐AEX clarified feed materials are shown in Appendix Tables A1 and A2, respectively.

The post run cycle is simply a single steady‐state cycle with the feed pump flow rate set to 0 and the “Feed” and “Flowthrough” positions switched to “Rest.” This allows for any antibody bound to the columns to be eluted and all of the columns to be equilibrated.

#### *Single Cycle PCC Runs*

Single cycle experiments were performed with the preload step, a single steady‐state cycle and the post run cycle. Elution fractions were manually collected from the elution outlet with every switch of the elution column position. Neutralization buffer was added to each elution fractionation tube: 2 mL for the depth filter clarified runs and 1 mL for the FT‐AEX clarified runs. Flowthrough fractions were manually collected for the preload step and with every column switch. Wash/Strip/Equilibration fractions were collected with every column switch.

#### *100 Cycle Experiments*

100 cycle experiments were performed with the preload step, 100 steady‐state cycles, and the post run cycle. Elution fractions were collected from the elution outlet with every switch of the elution column position. Neutralization buffer was added to each elution fractionation tube: 2 mL for the depth filter clarified run and 1 mL for FT‐AEX clarified run. Flowthrough and Wash/Strip/Elution fractions were collected every 12th cycle (cycles 1, 13, 25, 37, 49, 61, 73, 85, and 97) and the 100th cycle. Intermediate cycles were pooled. An additional flowthrough fraction was collected for the preload step.

### *Analytics*

#### *Protein G—Antibody Quantification*

Antibody quantification was performed using a HiTrap Protein G 1 mL column (GE) attached to an Agilent™ 1200 HPLC system (Agilent, Santa Clara, CA, USA) with 100 μL injection volume. A 10 min step gradient method was performed with a flow rate of 2 mL/min throughout. Sodium phosphate (20 mM pH 7.0) was used as the equilibration buffer with a step gradient to glycine‐HCl (20 mM pH 2.8) after 3 min for elution. The column was re‐equilibrated by a step change to sodium phosphate after 7.5 min. Antibody concentration was calculated from peak area at 280 nm based on a theoretical antibody extinction coefficient of 1.462 mL/mg/cm.

#### *qPCR—DNA quantification*

CHO HCDNA quantification was performed using Cygnus Technologies CHO Host Cell DNA kits (#D555T) (Cygnus Technologies, Southport, CA, USA) according to manufacturer instructions. Power™ SYBR™ Green Master Mix (Life Technologies, Paisley, U.K.) was used to make up the amplification reagent as recommended by Cygnus Technologies.

#### *ELISA—HCP Quantification*

CHO host cell protein quantification was performed using Cygnus Technologies CHO Host Cell Protein Kits (#F550) (Cygnus Technologies) according to manufacturer instructions.

#### *SEC—Aggregate Quantification*

Aggregate quantification was performed on selected samples using size exclusion chromatography on a Tosoh TSKgel UP‐SW3000 (30 cm) (Tosoh Corporation, Tokyo, Japan) with guard column. An Agilent 1260 HPLC (Agilent) was used with a 5 μL injection volume and UV detection at 280 nm and 320 nm. A 15 min isocratic method was used at a flow rate of 0.35 mL/min with a mobile phase containing 100 mM sodium sulphate and 100 mM sodium phosphate at pH 6.7.

## Results and Discussion

### *Analysis of single column runs to provide parameters for the PCC model*

**Figure 4:**
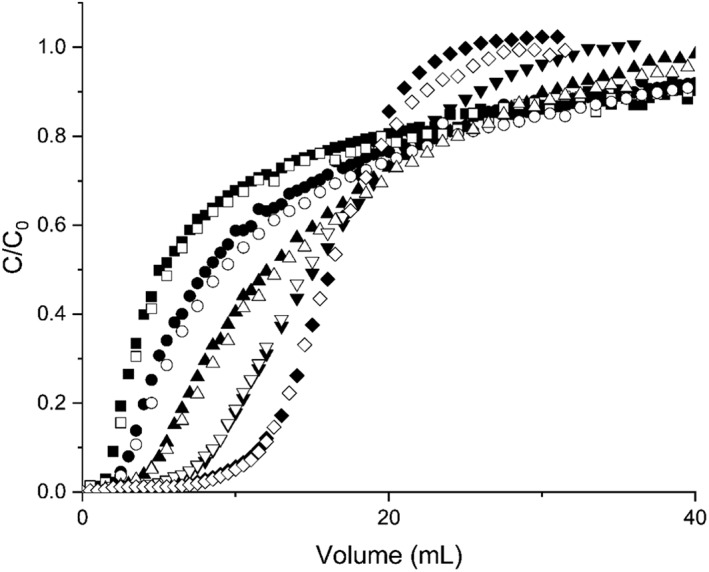
Breakthrough curves generated using depth filter clarified feed material at a loading residence time of 0.25 min (black squares), 0.5 min (black circles), 1 min (black triangles), 2 min (black upside‐down triangles), and 4 min (black diamonds) compared with breakthrough curves generated with FT‐AEX clarified feed material at a residence time of 0.25 min (white squares), 0.5 min (white circles), 1 min (white triangles), 2 min (white upside‐down triangles), and 4 min (white diamonds).

Figure 4 shows the breakthrough curves generated at different residence times on single 1 mL HiTrap MabSelect SuRe™ protein A column with both depth filter and FT‐AEX clarified feed material. As the residence time decreases, the breakthrough curve shifts to the left and the sharpness of the breakthrough curve diminishes as expected and has been recorded and explained by others in the literature.^21^ In the context of PCC, while lower residence time results in higher productivity, it also has the effect of reducing the usable capacity during PCC operation. This is because a larger proportion of the product flows through the column for more of the load volume, thereby resulting in greater preloading of the flowthrough column.

There is no significant difference in the breakthrough behavior of FT‐AEX clarified feed material compared to the depth filter clarified feed material, particularly at higher residence times. FT‐AEX clarified material has been shown to have significantly less DNA than material clarified through a standard two‐stage depth filtration train, and consequently less chromatin.^19^ Therefore, this result appears to contradict the findings of Gagnon et al. who reported that protein A dynamic binding capacity increased by 20% when chromatin depleted feed material was used to feed the protein A column.^22^ However, given that Gagnon focuses on batch conditions, and so does not go further than 10% breakthrough, it may be that, upon overloading the column, chromatin is competed off by the antibody. Alternatively, the contradictory performance may simply be due to differences in the feed material. For example, the material used in Gagnon's work was harvested at day 15–30 with viabilities of 20–50%. The material used in this work was harvested at day 14 with a viability of 64% or greater.

**Figure 5:**
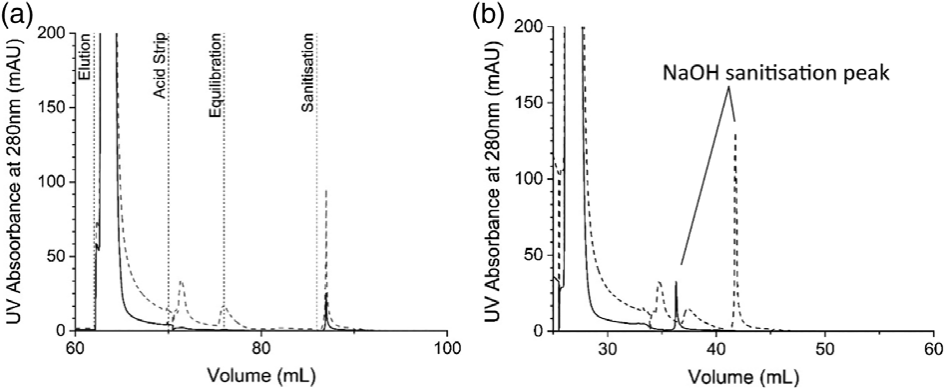
Figures showing regeneration steps performed during batch experiments to define conditions for the PCC runs. Graph (a) shows elution, acid strip, equilibration and sanitisation steps following a load of 40 mL with depth filter clarified feed material (dashed line) and FT‐AEX clarified material (solid line). Graph (b) shows the modified regenerations steps following a 20 mL load for depth filter clarified material (dashed line) and a 13.5 mL load of FT‐AEX clarified material (solid line). The non‐required acid strip and post acid strip equilibration were removed in this FT‐AEX clarified feed run.

A difference was, however, observed between the regeneration steps on columns loaded with FT‐AEX clarified material when compared to those loaded with depth filter clarified feed material. Figure 5a shows the elution, acid strip, equilibration, and sanitisation steps following loading with FT‐AEX clarified material compared to depth filter clarified material. The FT‐AEX clarified material results in a sharper elution peak, no acid strip peak, and a smaller sanitisation peak.

As a result of this finding, the regeneration for the FT‐AEX clarified material was shortened by reducing the elution to 5 column volumes and removing the acid strip while maintaining the re‐equilibration at 10 column volumes. As can be seen from Figure 5b, this shortened regeneration does not result in any significant change in the sanitisation peak for the FT‐AEX clarified material. As a result, these modified conditions were taken forward into the model and subsequent PCC runs.

### *PCC model predictions*

**Figure 6:**
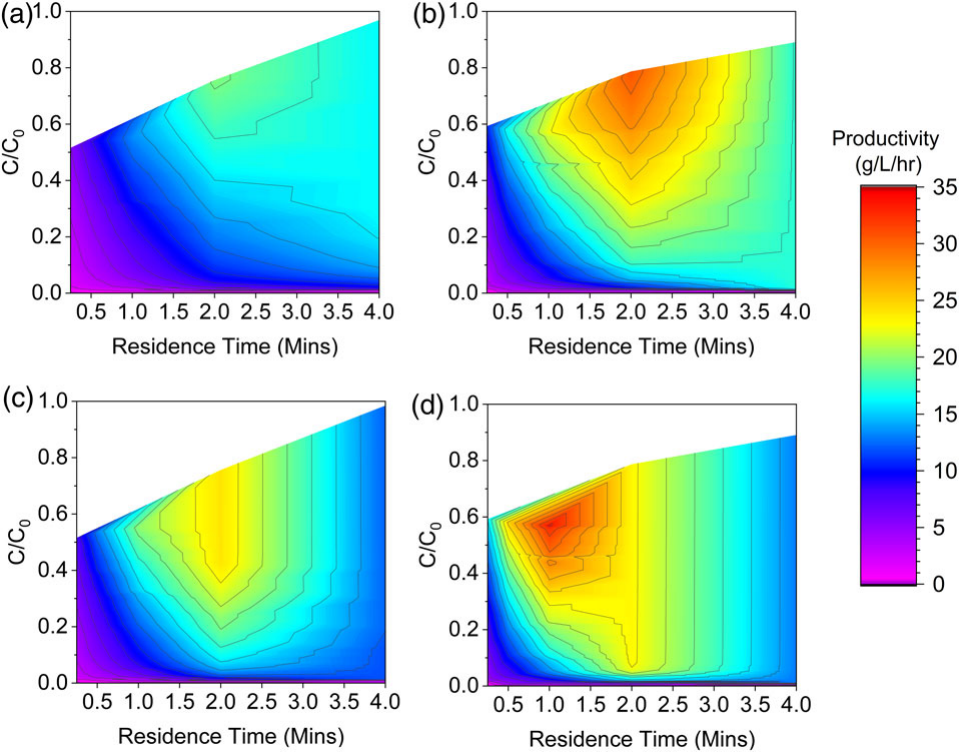
Predicted productivities using the mathematical model for a 3 column system with depth filter clarified feed material (a), 3 column system using FT‐AEX clarified feed material (b), a 4 column system with depth filter clarified feed material (c) and a 4 column system with FT‐AEX clarified feed material (d). These plots are based on the five residence times and use linear interpolation for intermediate values.

The experimental data from the breakthrough curves and optimized regeneration were applied to the model. Figure 6 shows the predicted productivities for a 3 and 4 column system using depth filter clarified material (a) and (c), respectively, and using FT‐AEX clarified material (b) and (d), respectively. The model predicts that for both 3 and 4 column systems the FT‐AEX clarified material allows for better productivities than depth filter clarified material. This is due to the ability to shorten the regeneration which provides better matching between load and regeneration steps for the three column system, and the use of lower residence times on a 4 column system.

The combination of FT‐AEX clarified feed material and a 3 column system was shown to achieve productivity gains of 28% over depth filter clarified feed material with a 4 column system. The reduction in the number of columns and increased productivity demonstrates chromatographic clarification as a useful tool to aid simplification of PCC.

A 4 column system at 1 min residence time with FT‐AEX clarified material gave rise to the highest predicted productivity. It is of note that further optimization is possible as the load was shorter than the regeneration by 4.5 min. By assessing performance at residence times of between 1 and 2 min, it would have been possible to further increase the productivity. However, the productivity gains predicted were thought sufficient as proof of concept.

### *Experimental validation of PCC model productivity predictions*

**Figure 7:**
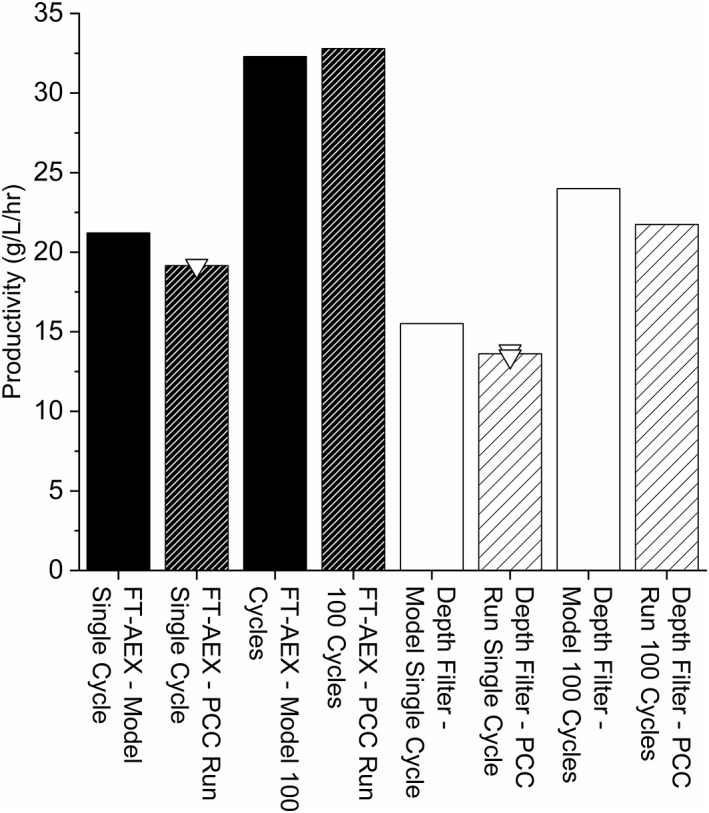
Productivities achieved experimentally versus those predicted by the model. The solid black bars show the predicted productivities for FT‐AEX clarified material and the dark hashed bars show the experimentally achieved productivities for FT‐AEX clarified material. The white bars show the predicted productivities for depth filter clarified material and the light hashed bars show the experimentally achieved productivities for depth filter clarified material. The (▿) show replicates of the single cycle runs.

The productivity predictions of the model were validated experimentally. Figure 7 compares the single and 100 cycle productivity predictions, for both FT‐AEX clarified feed material and depth filter clarified material, to the experimental results. The single cycle runs were performed in duplicate. There was no significant difference between the experimental productivities obtained. The 100 cycle runs were completed as a single replicate. Eluate titer measurements were taken for all four columns every 12 cycles. Excellent reproducibility was observed in eluate titers between the columns and across the cycles with an average mass of antibody eluted of 44.41 mg and a standard error of 0.34 mg for the depth filter clarified feed material and an average mass of antibody eluted of 32.98 mg with a standard error of 0.11 mg for the FT‐AEX clarified material.

The largest difference between predicted productivity and that achieved experimentally occurred for the single cycle PCC run with depth filter clarified feed material. This difference was 12.2%. This demonstrates good agreement between the model and experimental results and provides a similar level of agreement to the model proposed by Baur et al. The largest difference between the predicted and modeled productivity was 8.9% in their work.^10^

### *PCC performance with different feeds*

The PCC performance in terms of yield, HCP concentration, HCDNA concentration, and aggregates was measured at various points within the 100 cycle experiments for both the FT‐AEX and depth filter clarified material.

#### *Yield*

**Figure 8:**
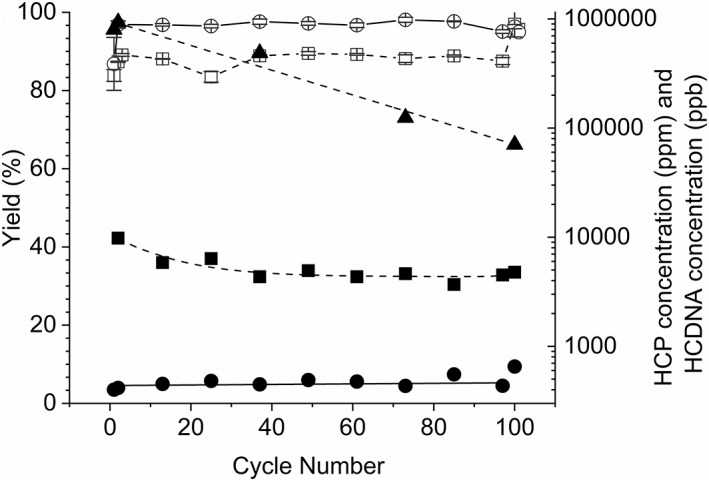
Graph showing yield, Host Cell Protein and Host Cell DNA concentration of the eluates versus cycle number for the 100 cycle PCC experiments. The yield (white squares) Host Cell Protein concentration (black squares) and Host Cell DNA concentration (black triangles) in the eluates from the PCC experiment using depth filter clarified material as the feed are compared to the yield (white circles) and Host Cell Protein concentration (black circles) in the eluates from the PCC experiment using FT‐AEX clarified material as the feed.

Figure 8 shows the average yield across all four columns versus cycle number. The yield is lower for the first cycle as this includes the initial preloading step. This preload is essentially passed from column to column during the run and is finally recovered in the post run regeneration steps. This is supported by the overall yield, in Table 7, which is approximately the same as the yield achieved from cycle 2 onwards.

**Table 7:** Key Performance Characteristics of the 100 Cycle PCC Experiments with Depth Filter and FT‐AEX Clarified Feed Material Compared to a Batch Run with a Normal Load, and Long Load Batch Runs which Were Loaded to the Same Degree as Their Respective PCC Runs. For Data Associated with the PCC Runs, the Standard Deviation is Presented in Brackets Below the Value. The Number in Square Brackets Underneath the Productivities Are the Percentage Increase in Productivity Compared to the Batch Run

	Depth Filter Feed Material Batch Normal Load	Depth Filter Feed Material Batch Long Load	FT‐AEX Clarified Feed Material Batch Long Load	Depth Filter Feed Material PCC	FT‐AEX Clarified Feed Material PCC
Load volume (mL)	9	20	13.5	15	10.5
Yield (%)	90.0	58.2	69.5	88.7 (4.05)	96.8 (4.19)
HCP (ppm)	3386	3184	266	5386 (1665)	476 (68.3)
HCDNA (ppb)	4.36 × 10^4^	2.26 × 10^5^	<8	4.82*10^5^ (3.46*10^5^)	<19.4
Percentage monomer (%)	Not measured	96.9	Not measured	95.9 (0.23)	98.3 (0.41)
Productivity (g/L/hr) [% diff to batch]	12.4	22.3 [+79.5%]	26.8 [+115.7%]	22.0 [+76.8%]	32.8 [+163.7%]
Relative capacity utilization compared to short load batch run	1.0	1.60	1.25	1.83	1.36
Difference between pre and post use column pressure drop (bar)	No significant change in pressure			0.30 (0.037)	0.10 (0.032)

The 100 cycle runs with depth filter clarified feed material do exhibit a lower yield than that of the FT‐AEX clarified feed material. This is due to the difference in operating conditions. In particular, the fact that more material was loaded on to the column for the PCC runs with depth filter clarified feed material. This would have resulted in the columns being more saturated causing the last column volume of feed material, before switching to post‐load wash, to contain more product. As this material would have been flushed to waste, this would cause the reduction in yield. This would also explain the lower yield compared to the batch short load (see Table 7). This lost material could be retained by washing on to next column in the loading zone, an approach that has been used by others.^23^

The lower yields displayed in Table 7 for the long load batch experiments, where the columns were loaded up to the same level of breakthrough as the PCC runs, are explained by the fact that there was no second column to bind unbound mAb in the flowthrough. As the FT‐AEX batch protein A run was loaded with less product, less product is lost.

#### *Host Cell Protein*

As observed in Figure 8, the HCP concentration in the FT‐AEX clarified protein A PCC eluates is at least one order of magnitude lower than that of the depth filter clarified protein A PCC eluates. In addition, the trends between the two challenges are different. The HCP concentration in FT‐AEX material remains constant or increases slightly whereas that of the depth filter material shows a downward trend.

It is hypothesized that this downward trend occurs as a result of fouling on the protein A column as there is no sanitisation step during the 100 cycle PCC experiment. Coelution of HCPs from protein A resin, as a result of binding to the resin rather than the product, has been shown to be minimal by Tarrant et al.^24^ but this work did not look at what remained on the matrix following elution. In contrast, Gagnon et al. looked at binding of chromatin to the protein A ligand and found that chromatin remained bound following elution and was responsible for leaching HCP contaminants into the eluate.^22^ As the cycles progress, the additional fouling of the resin with chromatin may reduce the leaching of HCP's from the column thereby reducing their levels in the eluate. Given that the FT‐AEX feed material contains much less DNA, and consequently much less chromatin, this would explain why HCP levels remain much more constant.

When comparing the HCP levels in Table 7, the batch long and short load show similar levels of HCP in the eluate while the PCC runs appear to have significantly more HCP. However, the ratio of HCP concentrations in the PCC eluates using depth filter and FT‐AEX clarified material is the same as the ratio of HCP in the eluates of the long load batch experiments. In both cases, the FT‐AEX clarified material gives rise to a HCP concentration approximately 11 times lower than that of the depth filter clarified material. Therefore, both batch and PCC experiments support the previous findings of Castro‐Forero et al.^19^ It is thought that the concentration of HCP is higher in the PCC eluates as the whole eluate was collected, whereas just the peak was collected for the batch experiments. If HCPs are leached during the elution then collecting just the peak will get rid of HCPs eluted outside of this peak, whereas collecting the whole eluate would not.

#### *Host Cell DNA*

Figure 8 shows the HCDNA concentration versus cycle number for the PCC eluates obtained using the depth filter clarified material. The DNA concentration versus cycle number for PCC eluates obtained using FT‐AEX clarified material are not plotted as these were below the limit of detection of the assay.

As with the HCP concentration, a downward trend in DNA concentration in the eluates for depth filter clarified feed material is observed with cycle number. By contrast to the reduction in HCP concentration, the reduction in DNA concentration does not level off. This suggests that DNA, probably in the form of chromatin, continues to bind to the column in increasing quantities as the cycles progress and the column becomes more fouled. This supports the hypothesis of the importance of DNA, and chromatin, in particular, with respect to column fouling.

Overall the level of DNA, in the PCC eluates, is 4.40 log lower for the FT‐AEX clarified feed material than that from the depth filter clarified feed material. A similar difference is seen in the long load batch results with the DNA in the protein A eluates from the FT‐AEX clarified material being 4.35 log lower than for the depth filter clarified material. Therefore, both batch and PCC experiments support the previous findings of Castro‐Forero et al. who found protein A eluates using FT‐AEX clarified material contained 3.5 log less HCDNA than protein A eluates from depth filter clarified material.^19^

#### *Aggregates and Low Molecular Weight Impurities*

Table 7 shows the average mAb monomer content, across cycle 2 and 100, in the protein A PCC eluates for depth filter clarified feed material and FT‐AEX clarified feed material. The long load batch run protein A eluate monomer content, for depth filter clarified feed material, is also shown. While the amount of product related impurities does appear slightly lower for the PCC eluates from the run using FT‐AEX clarified feed material, the difference is small. There are also slightly higher levels of product related impurities in the PCC run with depth filter clarified feed material, when compared to the long load batch run with the same feed material, and small decrease (from 96.2% to 95.7%) in the monomer content between cycle 2 and 100 for the PCC runs with this material. In contrast, the levels of monomer in the PCC eluates from the run using FT‐AEX clarified feed material where more consistent (98.3% and 98.4%, respectively). The small difference in the levels of product related impurities may be due to the fouling described in Host Cell Protein section of the Results and Discussion, which is consistent with the work of Lintern et al.^25^ who found a progressive reduction in the number of mAb and protein A fragments which were associated with the resin as the cycle number increased. However, due to the low magnitude of the differences and number of measurements taken, further investigation would be needed to draw firm conclusions for these differences.

#### *Productivity and Capacity Utilization*

Table 7 also shows the productivities achieved in batch and PCC mode for the different feed materials. The batch run with normal loading gave rise to the lowest productivity. As expected, the longer loading batch runs for both depth filter and FT‐AEX clarified feed materials resulted in a significant increase in productivity due to the additional loading, shorter residence time, and optimized regeneration conditions. However, as there was no column to catch product flowing through during overloading, the yield was adversely affected as discussed in the Yield section of the Results and Discussion.

As expected, the PCC runs resulted in the highest productivities. The PCC run with FT‐AEX clarified feed material resulted in the highest productivity which was 164% greater than batch and 49% better than the PCC runs with depth filter clarified material. This demonstrates that chromatographic clarification is a useful tool for enhancing the performance of protein A PCC for mAb capture. The increase in productivity seen between the long load batch runs and PCC runs is due to the fact that the regeneration steps happen in parallel with loading on other columns, for the PCC runs, reducing the impact of regeneration time on productivity.

The relative capacity utilization was highest for the PCC runs using depth filter clarified material. It was 83% higher than that of the normal load batch run and 35% higher than the FT‐AEX PCC run. This is because the load volume was greater and the residence time was longer. Capacity utilization with the FT‐AEX clarified feed could have been further improved by looking at residence times between 1 and 2 min as described in PCC Model Predictions section of this article.

It has been argued that maximizing capacity utilization is important for expensive resins.^10^ This is only true if the number of cycles that can be performed on the resin is constant, which may not always be the case. Pollock et al. showed that utilizing 100% of a resin's capacity can accelerate capacity loss.^15^ However, Mahajan et al. did not observe the same effect when a more alkali stable media was used up to 70% breakthrough.^26^ The difference between the findings of these two studies could be due to the higher loading in the first study and or the increased alkaline resistance in the second. In either case, the reduced fouling observed for the FT‐AEX PCC runs could allow less frequent sanitisation, and the reduced loading could allow the resin to be used for more cycles, both of which could reduce the impact of the lower capacity utilization.

#### *Post Use Pressure Drop and Column Discoloration*

**Figure 9:**
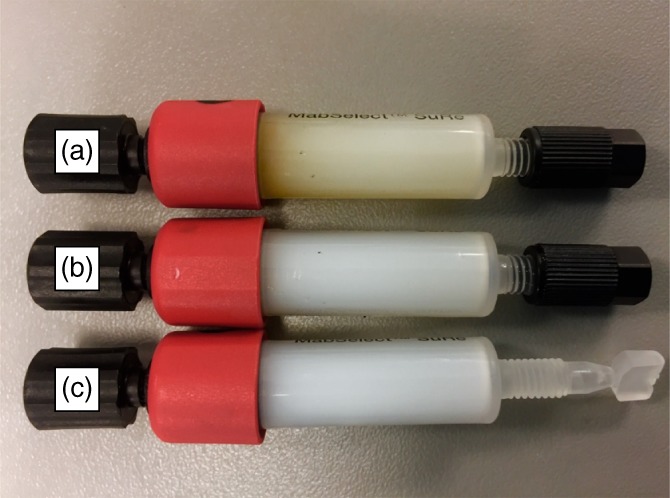
Picture of 1 mL MabSelect SuRe™ HiTrap Columns Post 100 PCC Cycles. Column (a) was loaded with depth filter clarified feed material, column (b) was loaded with FT‐AEX clarified feed material, and column (c) is a fresh unused column.

The fouling hypothesis discussed in the Host Cell Protein section, of the Results and Discussion, is supported by visual inspection of the protein A columns following 100 PCC cycles (Figure 9) which shows that the column exposed to depth filter clarified feed material is more discolored than the one fed with FT‐AEX clarified material.

The pressure drop across the columns following the 100 cycle PCC runs was also measured on an ÄKTA™ Avant using equilibration buffer. The pressure drop across a fresh column using the same buffer was measured and subtracted from the post PCC pressure. The results, in Table 7, show a significantly higher pressure drop for those columns on which the PCC was performed using depth filter clarified feed material, again supporting the conclusion of greater fouling on PCC columns loaded with depth filter clarified feed material.

## Conclusions

The research presented in this article has shown that chromatographic clarification is able to improve the product purity achieved during protein A PCC by reducing the level of HCP by 11‐fold and HCDNA concentration by 4.4 LRV when compared to PPC eluates from depth filter clarified material. This was similar to purity improvements demonstrated in batch processes.^19^ The levels of product related impurities were comparable between the second and last cycles of the FT‐AEX PCC runs, and comparable to the batch long load runs with depth filter clarified feed material, indicating that the performance of the PCC in terms of product related impurities remained consistent over the 100 cycles tested.

In addition, chromatographic clarification using the Emphaze™ AEX Hybrid Purifier was able to enhance the productivity of the protein A PCC by 49% compared to standard depth filter clarified material. This was due to the shorter regeneration, which allowed for shorter loading residence times to be used while maintaining the balance between loading and regeneration. The use of shorter loading residence time is the main driver for increased productivity. FT‐AEX clarified material also provides the option to reduce the number of columns from 4 to 3 with productivity gains of 28%, as predicted by the model presented herein. In addition, reduced fouling seen on the FT‐AEX PCC runs could provide the option for less frequent sanitisation, allowing more cycles to be completed per column with less capacity loss, and helping to drive down the cost of the protein A step.

Based on these results, it has been demonstrated that chromatographic clarification is a useful tool for enhancing the performance of PCC both in terms of purity and cost. By replacing the polishing grade depth filter, chromatographic clarification using the Emphaze™ AEX Hybrid Purifier can be easily incorporated into a batch or continuous process, in a scalable fashion, without adding additional separate unit operations.

## Appendix

**Table A1:** Step Times and Column Positions for the Depth Filter Clarified Feed Material

Switch. Step	Step Time	Column 1	Column 2	Column 3	Column 4
1.1	10	Feed	Flowthrough	Equilibration	PL wash
1.2	10	Feed	Flowthrough	Equilibration	Elution
1.3	6	Feed	Flowthrough	Rest	Elution
1.4	4	Feed	Flowthrough	Rest	Acid Strip
2.1	10	PL wash	Feed	Flowthrough	Equilibration
2.2	10	Elution	Feed	Flowthrough	Equilibration
2.3	6	Elution	Feed	Flowthrough	Rest
2.4	4	Acid Strip	Feed	Flowthrough	Rest
3.1	10	Equilibration	PL wash	Feed	Flowthrough
3.2	10	Equilibration	Elution	Feed	Flowthrough
3.3	6	Rest	Elution	Feed	Flowthrough
3.4	4	Rest	Acid Strip	Feed	Flowthrough
4.1	10	Flowthrough	Equilibration	PL wash	Feed
4.2	10	Flowthrough	Equilibration	Elution	Feed
4.3	6	Flowthrough	Rest	Elution	Feed
4.4	4	Flowthrough	Rest	Acid Strip	Feed

**Table A2:** Step Times and Column Positions for the FT‐AEX Clarified Feed Material

Step	Step Time	Column 1	Column 2	Column 3	Column 4
1.1	5	Feed	Flowthrough	Elution	PL wash
1.2	5	Feed	Flowthrough	Equilibration	PL wash
1.3	0.5	Feed	Flowthrough	Equilibration	Elution
1.4	4.5	Rest	Rest	Equilibration	Elution
2.1	5	PL wash	Feed	Flowthrough	Elution
2.2	5	PL wash	Feed	Flowthrough	Equilibration
2.3	0.5	Elution	Feed	Flowthrough	Equilibration
2.4	4.5	Elution	Rest	Rest	Equilibration
3.1	5	Elution	PL wash	Feed	Flowthrough
3.2	5	Equilibration	PL wash	Feed	Flowthrough
3.3	0.5	Equilibration	Elution	Feed	Flowthrough
3.4	4.5	Equilibration	Elution	Rest	Rest
4.1	5	Flowthrough	Elution	PL wash	Feed
4.2	5	Flowthrough	Equilibration	PL wash	Feed
4.3	0.5	Flowthrough	Equilibration	Elution	Feed
4.4	4.5	Rest	Equilibration	Elution	Rest
